# The art of balancing: the facilitator’s role in briefing in simulation-based learning from the perspective of nursing students – a qualitative study

**DOI:** 10.1186/s12912-020-00493-z

**Published:** 2020-10-22

**Authors:** Hilde Solli, Thor Arne Haukedal, Sissel Eikeland Husebø, Inger Åse Reierson

**Affiliations:** 1 Department of Nursing and Health Sciences, Faculty of Health and Social Sciences, University of South-Eastern Norway, Postbox 235, 3603 Kongsberg, Vestfold and Telemark Norway; 2 Department of Quality and Health Technology, Faculty of Health Sciences, University of Stavanger, Postbox 8600, 4036 Stavanger, Rogaland Norway

**Keywords:** Bachelor’s in nursing, Briefing, Facilitator, Nursing students, Prebriefing, Qualitative, Scenario, Simulation-based learning, Simulation-based training, Systematic text condensation

## Abstract

**Background:**

Facilitators plays a key role in nursing student’s learning when briefing them for simulation scenarios. However, few studies have explored the importance of the facilitator’s role in preparing students from the students’ perspective. The aim of this study was to explore undergraduate nursing students’ perspectives of the facilitator’s role in briefing.

**Methods:**

An explorative, qualitative approach was used. Four focus group interviews with a total of 30 nursing students constituted the data source. Data collection took place in December 2017 and in May 2018. The data was analysed using systematic text condensation.

**Results:**

Two main categories were identified: “The importance of framing the subsequent scenario” and “The importance of instructing students how to execute nursing actions in the subsequent scenario”. The first category consisted of three subcategories: providing predictability, providing emotional support and providing challenges. The second main category also consisted of three subcategories: providing information about medical and technical equipment, providing a demonstration of the monitor and providing a demonstration of the manikin.

**Conclusion:**

A briefing is more than a general introduction to a simulation scenario, learning objectives, roles, simulation environment and medical equipment. The information provided in a briefing is important for nursing students’ understanding of what they will encounter in the simulation scenario and what is being simulated, as well as possibly being a prerequisite for mastery. The facilitator’s role in the briefing is complex and requires a high level of educational expertise to balance the diversity of students’ learning approaches. Students have to learn how to simulate before the briefing. Therefore, we suggest separating the concepts of prebriefing and presimulation from the concept of briefing, introducing prebriefing and presimulation preparation before briefing, and possibly dividing students into groups based on their learning approach. Such interventions will make it possible for facilitators to balance between students’ needs and the time available for briefing.

**Supplementary information:**

**Supplementary information** accompanies this paper at 10.1186/s12912-020-00493-z.

## Background

Simulation-based learning (SBL) has become a widely used technique to prepare nursing students for practical placement [1–3]. It allows students to mimic both simple and complex clinical situations in a safe, structured and supported environment without harming patients [2, 4]. SBL is usually structured in three sessions: briefing, simulation and debriefing [5]. Briefing is an activity that takes place before onset of the scenario. In the literature, the concepts of briefing, prebriefing and presimulation are often used synonymously [5]. However, in this study we will use briefing.

Briefing is an important part of SBL as it prepares learners to understand what they are going to do and what they will be simulating, and it forms the basis for the debriefing [6–8]. To achieve optimal learning outcomes, a facilitator organizes and guides the SBL in a skilled and focused way [9–11]. The facilitator’s role is to prepare learners for the simulation, providing them with information about the scenario, the environment, the manikin, crucial medical equipment, and the need for mutual respect and confidence [5, 9, 10, 12]. A study by Husebø et al. [7] found that facilitators faced instructional challenges when trying to provide both instructions and demonstrations during the short briefing time. Although in every briefing the facilitator demonstrated the team leader’s position and how to use equipment, the nursing students misunderstood the right position to take in the simulation room and failed to master the use of the medical equipment in the simulation. This showed there was a difference between what it was possible to observe and do with a manikin versus a human being. It was crucial for the facilitator to clarify the similarities and differences between simulation and reality to make it easier for the students to achieve their learning outcomes [13–15].

Research has shown that nursing students experience high levels of stress related to simulation and worry about their lack of competence [16–19]. Therefore, it is important that facilitators demonstrate a positive attitude, calmness and trustworthy behaviour [9]. In addition, Jones et al. [14] list flexibility, familiarity with simulation as a teaching pedagogy and an understanding of how adult students learn as important facilitator characteristics. Facilitators also should be able to provide prompt feedback, establish a collaborative learning environment, and provide affirmation of simulation roles, in addition to having the practical knowledge necessary to demonstrate up-to-date clinical skills.

Although the facilitator plays a key role in the briefing, only a few studies have investigated how students see this role [18, 20]. Haraldseid, Friberg and Aase [20] found that nursing students felt it was important for facilitators to clarify the learning outcomes in SBL. The students needed detailed and structured information about the procedures in the scenario, as well as help understanding the whole picture of the scenario. Paige and Morin [18] explored nursing students’ perspectives on SBL. Concerning briefing, the students stated that facilitators should be familiar with all parts of the scenario, the equipment and the manikin, and the clinical procedures. They expressed that it was important for facilitators to treat the manikin like a real patient and to tell students what to do without using the word “pretending”, as the students performed the simulation just like an exam. They also wanted facilitators to review the simulation learning outcomes with them verbally. To reduce stress, the students wanted to presimulate to get more familiar with the manikin and the equipment beforehand. The students’ limited knowledge of the facilitator’s role in the briefing session speaks to a need for more research on this topic. The aim of the current study was to explore nursing students’ perspectives on the facilitator’s role in briefing in SBL.

## Materials and methods

### Design

We applied an explorative descriptive design, as it allows an open approach and is well suited to projects that aim to explore participants’ experiences and preferences. In addition, explorative design is recommended when research on a given phenomenon is scarce [21].

A qualitative approach using focus groups interviews (FGI) was applied to collect the data [22–24]. With focus groups (FG), participants have the possibility to discuss and consider each other’s point of view and thus provide rich data in their dialogues. The researchers also have the possibility to ask participants additional questions, thereby exploring arguments raised in the FG discussion in more depth. The data from the FGI was analysed using systematic text condensation (STC) [25].

### Participants

A purposive sample was chosen because the university had two cohorts of nursing students from a bachelor’s programme in nursing taking the same simulation course [23, 24]. In total, 137 students (83 full-time students and 54 part-time students) attended the same mandatory simulation course in their second year. All students in both cohorts were invited to participate in the study. A total of 38 students (24 full-time students and 14 part-time students) gave written consent before they were enrolled in the study.

### Setting

The compulsory simulation course took place at a simulation centre in a university in the south-eastern part of Norway, prior to the nursing students’ clinical placement in acute and critical nursing care. The simulation consisted of six scenarios focusing on deteriorated patient situations in the hospital setting. The learning outcomes included assessing airway, breathing, circulation, disability and exposure (ABCDE) [26]; prioritizing relevant nursing actions; and executing effective communication and clear leadership. Three high-fidelity simulators, one SimMan® and two ALS®, were used.

All students participated in a two-day simulation course. The students were split into 15 groups of 7 to 11 participants. Two students in each scenario acted as nurses, while the rest observed. The observers received a structured observation form which included the learning outcomes. Each scenario comprised the simulation activities and lasted for two hours. The briefing session lasted for 30 min in the first scenario and 15 min in the following five scenarios. Six nursing faculty employed at the university facilitated the briefing sessions. All facilitators had completed a three-day facilitator course, had several years of experience as facilitators, and were familiar with the structure of the simulations, the manikin and the technology used.

Prior to the simulation, the students watched a video regarding the function of the manikin, in addition to receiving written and oral information about the content and the organization of the scenarios. Descriptions of all the scenarios with references to relevant literature were accessible via a digital learning platform. As a stimulus for learning, the students could voluntarily answer a digital questionnaire with multiple choice questions regarding pathophysiology, symptoms and nursing actions associated with each of the scenarios. The students received individual electronic feedback on the correct answers to the questions.

### Data collection

The data collection took place in December 2017 (full-time students) and May 2018 (part-time students). The research group developed a semi-structured interview guide through a process that spanned several meetings. The guide was inspired by the NLN Jeffries Framework for Simulation [10, 12, 27] and previous research on the facilitator role [14] and briefing [3, 6, 7, 28]. A pilot FGI was conducted with two of the full-time students who had given written consent. The pilot showed that the first question in the guide was superfluous, as the substance was covered by other questions [24]. This question was removed from the guide. The interview guide used in current study has been uploaded as a supplementary file. The pilot FGI served as a check of the interview process and was not included in the data analysis.

In order to capture the students’ immediate simulation experiences, the four FGIs (two with full-time students and two with part-time students) took place directly after the students’ last simulation. Thirty of the 38 students who had signed a written consent form participated in the FGIs, as six students did not show up at the time when the FGIs were to start and two of students already were enrolled in the pilot FGI. Table 1 shows an overview of the distribution and characteristics of participants in the FGIs. The current study was a part of a more comprehensive study exploring students’ perspectives on the facilitator’s role in SBL; this study is about the briefing part of SBL. The FGIs addressed all three parts of SBL and lasted each for 60 to 90 min.

**Table 1:** Distribution and characteristics of participants in the FGIs

Focus group	Participants (no.)	Full-time students: 3 years (no.)	Part-time students: 4 years (no.)	Female/male (no.)	Age (range)
**1**	9	9		7/2	20–39
**2**	8	8		6/2	20–38
**3**	6		6	5/1	21–25
**4**	7		7	7/0	22–40

The four FGIs were moderated by four faculty at the university and the third author, none of whom facilitated the course. These individuals were chosen because they were available at the time the FGIs had to be executed. They were all registered nurses: two professors, two associate professors and one assistant professor. All moderators were skilled in moderating FGIs [24] and two were also skilled SBL facilitators. Prior to the FGIs the moderators were informed by the first author about the aim of the study, the interview guide, the simulation course, and the students’ learning outcomes. In addition, they observed a couple of scenarios executed in the simulation centre [29]. The FGIs were recorded and no field notes were produced.

### Ethical consideration

The study was approved by the Norwegian Centre for Research Data (NSD) (ref. no. 56123) and the dean. All the participants received oral and written information about the study, confidentiality, voluntary participation and their right to withdraw from the study at any time [30].

### Data analysis

The data were transcribed verbatim by an agency and analysed using STC [25]. All authors, contributed to the analysis. All were registered nurses, skilled in SBL and qualitative research methods, who worked as academics at the university, three of them as associate professors and one as a professor.

In the first step, the authors read the interviews in an attempt to get an impression of the whole. Discussion of the text gave rise to the identification of four preliminary categories, namely structure, information about the scenario, information about the equipment and expectations. In step two, the authors organized the data into meaning units. Then the first author sorted the meaning units into codes and sorted the codes into the preliminary categories. Through an iterative process, the first author revised the categories. In step three, the first author established sub-categories within the categories and condensed the content in each category. The condensates were written in the first person to ensure they corresponded with the students’ statements [25]. Illustrative quotations were added to the condensate. As their understanding of the results evolved, the authors reconceptualized categories and sub-categories. The authors discussed the results in relation to existing knowledge on the topic. In the fourth step, the condensates were synthesized in an analytic text with illustrating quotations. Table 2 shows excerpts of the analysis process.

**Table 2:** Excerpts of the analysis process

**Preliminary categories** **Step 1**	Structure, information (scenario), information (technical equipment), expectations
**Category** **Step 2**	Framing the group
**Sub-category** **Step 2**	Providing predictability
**Condensate** **Step 3**	We got sufficient briefing about the background and the patient in all the scenarios and we did not miss anything. We were pre-informed, so the briefing was short, but not too short. Those were the most important things that were addressed. The briefing was a “brushing up” on what we were prepared for, so this was nice. It was the summary of the scenario. It was very satisfactory that the facilitator gave us little information but provided tips and hints about what we had to focus on. We got some “threads” that helped us to master the challenges. It’s nice and easy to get information up front to know what to do before it happens. The information about the learning objectives in the briefing focused our learning.
**Analytical text** **Step 4**	Most of the students felt the scenario contents were sufficient and that they did not miss anything. The most important things were pointed, out and it was just a brushing up with some hints and tips related to what they needed to focus on. A student stated, “Very good that the facilitator gave just some info, with ideas and hints about what we needed to consider” (student FG 1). The tips and hints provided the students a feeling of having a toolbox that helped them to do their best in the simulation session, “[It is] an absolute advantage to receive the information beforehand, as one then has the possibility to know how to deal with a situation before it happens” (student FG 2).

The authors’ final analytical discussion ensured the internal consistency of the content, quotations, sub-categories and categories. Table 3 shows the steps in the data analysis.

**Table 3:** Examples of steps in the data analysis

Meaning unit	Sub-category	Category
“Very good that the facilitator gave just some info, with ideas and hints about what we needed to consider”	Providing predictability	The importance of framing the subsequent scenario
When I went in, I had “COPD” and I had to ask myself “Do I actually know anything about COPD? What am I supposed to do, and which actions need to be taken? Then we went out with the facilitator who provided a resumé of the situation, saying “you can do this or this”. This was very pleasant, and it really helped.	Providing emotional support	
I liked not having received all the information up front. When we started the scenario, we didn’t really know what was behind the door. It meant that we had to observe the clinical signs and take it from there. I liked this a lot, because I wouldn’t risk concentrating on other things when I knew that a cardiac arrest was about to happen. These “surprise elements” were valuable and made my learning process better.	Providing challenges	
It was good to know where things were in the simulation room. Looking back, it wasn’t always so clear how we were supposed to use it, but we were told during the simulation; at least that was my experience.	Providing information about medical and technical equipment	The importance of instructing students how to execute nursing actions in the subsequent scenario
They could have given us better information about the monitor, how to use it and enter the numbers. In some of the scenarios, the old values were there and in others we had to start it ourselves and it was a bit … A lot of us didn’t know how to get the values on to the monitor. They could have made this clearer since we spent a lot of time, which could have been used doing other things.	Providing a demonstration of the monitor	
I would have liked more information about the manikin before the simulation. I know that several of us felt that we hadn’t received enough information prior to the simulation. For example, the facilitator told us that the manikin could cry and vomit and so forth. But during the actual simulation this was only by sound! Maybe some of us expected that liquid or something was supposed to come out.	Providing a demonstration of the manikin	

### Rigour

The trustworthiness of the study is considered in terms of its credibility, dependability, confirmability, transferability and authenticity [29].

We have tried to enhance credibility by giving a detailed description of the design, the participants, the setting, the data collection and the iterative analysis process to show how well these processes addressed the intended aim of the study and provided internal validity. To enhance dependability, we have described how we executed a pilot FGI exploring whether the interview guide was useful in fulfilling the aim of the study. The supplementary file shows the adjusted interview guide used in the FGIs. None of the authors who facilitated the course conducted the FGI; this was done to avoid the possibility that students might respond differently to the persons who were in charge of evaluating them on an upcoming exam. To establish confirmability, we have described the analytical process in which all the authors discussed labels, categories and sub-categories repeatedly in several meetings until agreement was achieved. In this way, we emphasized objectivity and ensured that the data represented the information given by the informants. To promote transferability, we have presented a rich description of the participants, the location, the setting and the analysis. To enhance authenticity, we have explained why we chose the informants as we did. We have described in detail the simulation course the students attended and the kind of information they received beforehand.

In addition, we used the Consolidated Criteria for Reporting Qualitative Research (COREQ), a 32-item checklist for interviews and focus groups, to confirm the quality of the methods section [31].

## Results

The analysis resulted in two main categories: 1) the importance of framing the subsequent scenario, consisting of the three sub-categories providing predictability, providing emotional support, and providing challenges; and 2) the importance of instructing students how to execute nursing actions in the subsequent scenario consisting of the three sub-categories providing information about medical and technical equipment, providing a demonstration of the monitor and providing a demonstration of the manikin. Table 4 displays the categories and sub-categories. We elaborate further on these findings below. The quotes are numbered 1 to 4 according to the FGIs.

**Table 4:** The categories and sub-categories

Categories	Sub-categories
The importance of framing the subsequent scenario	- Providing predictability - Providing emotional support - Providing challenges
The importance of instructing students how to execute nursing actions in the subsequent scenario	- Providing information about medical and technical equipment - Providing a demonstration of the monitor - Providing a demonstration of the manikin

### The importance of framing the subsequent scenario

The first main category highlights the importance of how facilitators balance students’ diverse needs for mental support, including improving their preparedness for the subsequent scenario and providing challenges.

### Providing predictability

The extent to which the students had prepared for the simulation varied. In addition, some students had not received the information provided by faculty in class prior to the simulation course. This varied level of preparedness for the simulation created uncertainty among the students: *“We got information about this beforehand and some [students] were there on the day and some weren’t, so it created a lot of nervousness and speculation about what to do” (student FG 3).* During briefing, the facilitators provided the students with opportunity to raise their concerns regarding the simulation, and generally the facilitator provided satisfactory answers. However, some students mentioned that it was difficult to ask relevant questions at first, since they did not know what was going to happen in the subsequent scenario:

*We did have the opportunity to ask questions, and we received answers to the simple questions we asked in order to be better prepared. For every new situation, our questions became more and more specific since we learned what to ask about*. *(student FG 4).*

This quotation might reflect the students’ experience of progress in their learning process during the briefings. This back and forth of questions and answers seemed to be a dialectic process in which students gradually developed competence in focusing on the most crucial information.

However, most of the students considered the information regarding the scenario to be sufficient and felt they had not missed any important information. The facilitator had pointed out the most important matters they needed to keep in mind. The briefing was a refresher with some hints and tips related to what they needed to focus on in the scenario. One student stated, *“[It was] very good that facilitator gave just some info with ideas and hints about what we needed to consider” (student FG 1).* The information presented provided the students with a toolbox that helped them to create a mental picture of the subsequent scenario: *“[It was] an absolute advantage to receive the information beforehand because then you know how to deal with a situation before it happens” (student FG 2).* Some students also mentioned appreciating the focused information the facilitators provided to the observers: *“[It was a] positive [thing] that the observers knew what to look for, so this provided ideas [that were] absent at the outset” (student FG 1).* The students’ descriptions display how important it was for facilitators to provide specific information, tips and answers in a way that created a sense of predictability with regard to the subsequent scenario.

### Providing emotional support

The results demonstrated that the students experienced varying degrees of emotional support from the facilitators. Some students underscored the facilitators’ important role in acknowledging student perspectives and showing empathy and understanding regarding their anxiety about the scenario. They characterized the facilitators as calm, and this calmness influenced the students’ level of stress. The facilitators provided emotional support when they told the students that they themselves had felt unprepared when attending simulation courses and therefore recognized the insecurity and stress:

*I have to emphasize that both we and our facilitators underwent the same experience. They had just attended a simulation course. They had to practice, and they got the feeling of stress and insecurity as well. It was good to know that we shared the same unsettling situations as the facilitators. (Student FG 3).*

However, other students emphasized that their stress levels were affected by how the facilitators presented information. If the facilitators provided a large amount of information in a short time, the students experienced the situation as stressful and found it difficult to remember what to do in the scenario:

*We had half an hour with a good deal of information. And I, for one, get stressed when I am in this sort of situation with fellow students. When it wasn’t my turn, I hardly remembered anything when I had to start …*. S*o I think there could have been more clarity or even better information in order to avoid the nervousness and uncertainty about what was supposed to happen. (Student FG 3).*

The students also explained that how facilitators addressed little-known procedures could influence emotional stress. This was the case for scenarios that included calculation of medication dosages. At this stage in the students’ education, there had been little focus on this skill. Therefore, they questioned why the facilitators handled this topic very differently. In one scenario, the facilitator showed students how to calculate dosage, which alleviated their stress. In another scenario, the facilitators provided very little information about dosage. The students, who were stressed about giving medication, wanted demonstrations of how to calculate dosages in the briefing and not during the simulation or in the debriefing. One student commented, *“Information in relation to medication management was deficient, and this lack of knowledge made me insecure on a number of levels” (student FG 3).* In this case, the students stated how important it was for reducing their stress that all the facilitators gave them the same thorough review of little-known procedures.

In general, most of the students considered that they got adequate emotional support. However, some students were upset about being put in scenarios that they felt surpassed their own level of competence. In such situations, they experienced a lack of emotional support and understanding of what kind of preparation they needed beforehand. They wanted to be fully prepared for such situations and not be pushed beyond their limits in the actual simulation: *“It was an unpleasant experience to drop into the scenario with cardiac arrest. This is serious, and I want to be trained in everything for such situations beforehand” (student FG 1).* These comments suggest how important it is for students that facilitators acknowledge their anxiety and responding to them with a demeanour that encouraged stress reduction.

### Providing challenges

Some students mentioned the importance of being given the opportunity to master unforeseen events. They reported having experienced some unexpected moments during the scenarios for which they could not have been prepared. One student stated, *“[It was] more educative when we lacked full information, but surprises cropped up now and then” (Student FG 1).* Such unexpected moments created challenging learning opportunities: *“The lack of information and training in the briefing means that you have to solve it in the situation, and you will therefore remember it next time around” (student FG 4).*

Unexpected situations were compared to clinical situations, as in the following remark: “*When such surprises happened, it gave me an experience of actually being out in practice, because unplanned things can happen there. Monitor values can decrease suddenly, so when things suddenly occur, it is a touch of reality” (student FG 1).* Hence the facilitator played an important role in challenging the students and giving them the opportunity to master unforeseen events like the ones they will encounter in practical placements.

## The importance of instructing students how to execute nursing actions in the subsequent scenario

The second main category describes the importance of facilitators’ balancing students’ different needs for practical information and demonstrations of the equipment in simulation room, the monitor and the manikin to improve their chances of carrying out adequate interventions in the simulation.

### Providing information about medical and technical equipment

The results demonstrated that the students felt the facilitators briefed them well concerning the functionality of the simulation room, including where they could find necessary medication and medical technical equipment. However, the students found the information regarding some of the equipment unclear:

*Having to manually measure blood pressure spoiled things a bit for us. We stuck to that blood pressure and saw that the level hadn’t changed, so we thought that this was OK for the patient, which in fact it wasn’t. And we hadn’t got the information we needed on the touch-screen when you are going to measure a new blood pressure. (student FG 3).*

Some students similarly pointed out their need for more demonstration of a specific ventilation bag, while some mentioned wanting to be trained in the use of all the equipment they might encounter prior to simulation:

*Giving us such an important scenario as cardiac arrest, I feel that we should have been trained in absolutely everything, not thrown into it. After all, it is a life-saving situation, so I would have wanted to be able to master everything accurately before simulating it. (student FG 1).*

This comment exemplifies how important some students perceived the facilitators’ demonstrations of relevant medical and technical equipment to be in enabling them to achieve their learning outcomes.

### Providing a demonstration of the monitor

Some students expressed satisfaction with how systematically the facilitator explained and demonstrated the function of the monitor: *“The systematic demonstration of the monitor, which we are not used to, was very satisfactory” (student FG 2).* Responses from the students showed a differentiation between those who were satisfied with the demonstration provided and those who needed a more extensive demonstration of the monitors. Moreover, some students also stated that it was not possible to remember all of the information presented. In addition, they reported that the monitor was used differently in different scenarios and therefore wanted the facilitator to provide more extensive information and a better demonstration before the first scenario: *“At the outset, it was a case of ‘Where [on the monitor] is the pulse, where is it?’ To dampen the somewhat chaotic feeling, the first group could have received a little more demonstration” (student FG 4).* Because the monitor was used differently in each scenario, it was important for the facilitator to adjust the information and demonstration from the previous scenario. The review of the monitor in the very first scenario was of particular importance.

### Providing a demonstration of the manikin

The students differed in their needs for a demonstration of the manikin’s features. Some were satisfied with the facilitators’ demonstration of how they could communicate with the manikin and with instruction regarding what they were not allowed to do with the manikin: *“They [the facilitators] provided very clear information regarding the fact that we were not to inject anything into the manikin … So that was very reassuring” (student FG 1).* Other students mentioned that the information facilitators provided about the manikin and their demonstration of its special effects were insufficient. Students felt uncertain about how to palpate the manikins’ pulse and even about whether the manikin actually could vomit or just produce a vomiting sound:

*I know that there are many who felt that we had got too little information ahead of the simulation. Because they talked about ‘yes, the mannequin can cry, it can vomit’ and the like, but during the simulation this happened aurally; there were perhaps some who expected to see fluid coming out or something, for example things like that. (Student FG 1).*

Students revealed different understandings or recollections of what information they received or what was demonstrated related to the use of the manikin. This finding also points to the importance of the facilitator’s role in exposing misunderstandings and adjusting to students’ requests.

## Discussion

The results of this study provide valuable insight into the fact that briefing is critical for nursing students’ understanding of what they will encounter in a simulation scenario. It might also be a prerequisite for students’ mastery of the scenario. The findings highlight the complexity of the facilitator’s role in briefing. In the following, we will discuss this complexity, as well as the possibility of re-thinking how briefings should be conducted in light of limited previous research.

It was a balancing act for facilitators to provide information that was as detailed as the more stressed students expected to receive while keeping some aspects of the scenario a surprise for the students who appreciated challenges. The diversity of nursing students’ perspectives on simulation design were explored by Paige and Morin [18], who identified different perspectives that facilitators should be aware of: the “Stand by me” perspective of students who want structure and guidance and prefer to work together with peers to reduce their own anxiety and the “Let me show you” perspective of students who want to figure things out by themselves with minimal guidance from the facilitator. Our findings also identified different attitudes and approaches towards learning and different expectations for how detailed the information presented by the facilitator during briefing session should be.

According to the NLN Jeffries Simulation Theory [10, 12, 27], facilitators provide crucial information about what students are supposed to do in a simulation. The students also pointed out the importance of being able to raise questions [10]. Simulation design is based on the expectation that students will prepare beforehand [10]. Facilitators are also expected to prepare to be able to properly guide the simulation [9, 10, 14]. In the current study, the variation in the students’ preparedness was challenging for the facilitators who had to find the right balance between providing adequate information for the unprepared students while simultaneously ensuring that the prepared students did not experience their own preparedness as unnecessary. The students expressed the importance of keeping to the time schedule for the briefing activity, which is also mentioned in the NLN Jeffries Simulation Theory [10]. However, given the amount of information that must be provided to meet the diverse needs of the students in only about 15 min (30 min for the first simulation), it might be difficult to meet the students’ different needs for information and demonstration.

Acting in the role of nurse leader or nurse assistant in SBL meant that the students had to make themselves vulnerable to the rest of the group, the operator and the facilitator. For many students, this position seemed to be extremely stressful, as also found in previous studies [16–18, 32, 33]. Boostel et al. [17] found that after participating in a high-fidelity simulation scenario, nursing students experienced stress connected to factors such as lack of competence, fear of causing psychological damage to the patient, not knowing how to respond to patients, seeing a patient die, encountering a situation without knowing what to do, relationships with colleagues (nursing students) or meeting a patient who has difficulty communicating [17]. Several of these factors were identified in the current study. Some students reported having difficulty remembering what the scenarios were about and what to do in scenarios involving patients with severe illnesses. Tyerman (2016), referring to simulation studies, reports that a high amount of stress was a hindrance to achieving learning outcomes. Jeffries [10] states that having some anxiety in the learning process, as in a competition, might stimulate the learners’ motivation to learn. However, when it comes to SBL, this is not recommended because too high levels of stress have been shown to impede mastery among learners. One of the responsibilities of a facilitator is to create a safe and secure learning environment for students, which includes reducing stress. Simultaneously, the facilitator should also challenge students to push their limits and master unexpected situations, which might lead to a sense of self-efficacy and better learning outcomes [10]. Several studies have shown that experiencing stress in simulations is a positive indicator for mastering stress in clinical placement [32, 33]. Recognizing the extent to which individual students are able to master stress is a challenge for facilitators, who are supposed to sense each student’s limit of tolerance in a limited amount of time.

We can divide the students in the current study into two groups based on how they perceived their educational needs: one group with the “Stand by me” perspective and another with a perspective more similar to “Let me show you” [18]. To meet these different needs in a student group in the space of 15–20 min seems challenging. The students in our findings expressed that the faculty ought to re-think simulation design. An implication for further practice might be to group students with similar learning perspectives together and provide them more tailored briefing sessions in terms of time, degree of detailed information provided and respect for the desire to be challenged [18]. This proposition raises questions such as, Would group composition have any influence on the amount of stress? Will students reach learning outcomes quicker or perhaps master challenges with more self-efficacy?

It was a balancing act for facilitators to accommodate all students when some of them preferred to be trained in using specific equipment while others were satisfied with review and demonstration of the equipment, as described in the second main category. It is vital to reflect on whether the students thought of the simulation as an exam or as a kind of training, like clinical scenarios without risk. Such pre-understandings might influence student outcomes. Rystedt and Sjöblom [8] note the importance of participants’ understanding what is actually being simulated in a simulation. This means students have to understand the ways in which a simulation is similar to clinical practice and the ways in which it is different. In the present study, one of the students seemed to recognize a similarity with clinical practice when the monitor values decreased. Another student described their experience of a cardiac arrest scenario as though it was a real situation and not a simulation of a situation that they could encounter in clinical practice. As Rystedt and Sjöblom [8] point out, it is important that students learn to focus on the goal of a simulation and not mix in irrelevant aspects that suddenly emerge, which will distract from this aim. Therefore, students must learn to simulate effectively if they are to achieve learning outcomes [8]. There are several perspectives on how to learn to simulate. Cummings and Connelly [34] found that repeated simulation experiences led to an increase in student confidence and active learning. Stephenson and Poore [28] claim that students should have the opportunity to play with the equipment before simulation to fully understand its functionality. Furthermore, Alfes [35] suggests implementing simulation activities at the beginning of nursing students’ education to increase student confidence with this kind of exercise.

The current study has described the diversity of students’ expectations and perceptions of the facilitator’s role in the briefing. For some students, the information and demonstrations provided by the facilitators met their expectations while for others, they did not. In light of this, one can discuss whether separate briefings should be offered. As described in the introduction, the concepts of prebriefing, presimulation and briefing are often used synonymously [5]. There are studies, however, that separate these concepts and integrate prebriefing and presimulation preparation as a separate concept in simulation design [3, 36]. Tyerman et al. [36] suggest separating presimulation preparation and prebriefing from briefing. Presimulation preparation and prebriefing activity should be related to reviewing knowledge and skills before a specific scenario, as this can help reduce students’ stress and promote the achievement of learning outcomes. Page-Cutrara [3] conducted a concept analysis of prebriefing. The literature review supported the idea that briefing should be separate and comprise functional and operational information immediately before the onset of the simulation. Based on the literature, the concept of prebriefing must support learning, and the facilitator’s role will be to provide information and training activity tailored to students’ level of knowledge, learning needs and former experiences of SBL [3]. Jeffries [10] suggests that students might have a presimulation activity for the purposes of training and getting to know the different equipment and manikins before the onset of the simulation activity.

Several students in current study stated that it was important that the facilitator provide them with more demonstrations of the medical equipment. They wanted to have the possibility to train with medical equipment and specific procedures before they were vulnerable in the simulation. Including prebriefing and presimulation preparation in simulation design would give students the opportunity to learn to simulate and probably improve their learning outcomes with less stress.

### Limitations

There are several limitations to this study. A purposive sample with students from one nursing education programme from one university was used. The results might therefore be somewhat more one-sided compared than if nursing education programmes from several universities were included. The unlike compositions of the moderator teams might have resulted in varying amounts of rich information from the different FGIs. The number of citations reproduced from each FGI might therefore be unbalanced. Due to practical considerations, the results were not returned to the participants to confirm our interpretation of the analysed data [31].

## Conclusion

The findings show that in SBL, the briefing is more than a general introduction to the simulation scenario, learning objectives, roles, simulation environment and medical equipment. The information provided during briefing is critical for nursing students’ understanding of what they will encounter in the simulation scenario and what is being simulated, as well as possibly being a prerequisite for mastery. The findings show that the facilitator’s role in briefing is demanding and complex and requires both a high level of expertise in the simulation technique and the ability to balance students’ diverse preparation needs. In the short time allocated for briefing, the facilitator has to simultaneously sense and adapt to the students’ varying levels of mental capability and preparedness.

To address the differences in students’ perspectives on the facilitator’s role in briefing, we suggest that students be divided into groups based on their learning approach. There is a need for simulation technique to be taught before students begin SBL, and we suggest that prebriefing and presimulation preparation could therefore be separated from the concept of briefing. These interventions could make it easier for facilitators to balance the needs of students and provide realistic opportunities to meet these needs.

The findings in the current study might be of interest for re-thinking the organisation of SBL in nursing bachelor’s education as well as in educational programmes for nurses as part of their professional development.

## Supplementary information

**Additional file 1.** The interview guide. COnsolidated criteria for REporting Qualitative research (COREQ Checklist).

## Data Availability

The datasets generated and/or analysed in the current study are not publicly available due to the sample and the importance of preserving the anonymity of the participants.

## Abbreviations

FG: Focus group
FGI: Focus group interview
SBL: Simulation-based learning
