# Supplementary material for: The art of balancing: the facilitator’s role in briefing in simulation-based learning from the perspective of nursing students – a qualitative study
Source: BMC Nurs. 2020 Oct 22;19:99. doi: 10.1186/s12912-020-00493-z (PMC7579795; doi:10.1186/s12912-020-00493-z)
Supplement: Supplementary file 1 — Additional file 1. The interview guide. COnsolidated criteria for REporting Qualitative research (COREQ Checklist). [file 12912_2020_493_MOESM1_ESM.docx]

**The interview guide**

We want to ask you some question about how you perceive the importance of the facilitator’s role in the briefing session in SBL.

- In what way did you experience the importance of the facilitator’s role in the briefing session in relation to your preparation for the simulation activity? [Explain how we define the briefing session.]
- How did you experience the facilitator’s briefing regarding

1) the scenario?

2) medical equipment in the simulation room?

3) the manikin?

- Did you have the opportunity to pose questions during the briefing?
- Did the facilitator respond to your questions properly?
- Do you have any thoughts on how the briefing could have better prepared you for the simulation?
